# Bacterial profile of ocular infections: a systematic review

**DOI:** 10.1186/s12886-017-0612-2

**Published:** 2017-11-25

**Authors:** Mebrahtu Teweldemedhin, Hailay Gebreyesus, Ataklti Hailu Atsbaha, Solomon Weldegebreal Asgedom, Muthupandian Saravanan

**Affiliations:** 1 Department of Medical Laboratory Sciences, College of Health Sciences, Aksum University, Aksum, Tigray Ethiopia; 2 Department of Public health, College of Health Sciences, Aksum University, Aksum, Tigray Ethiopia; 3 Tigray Health and Research Laboratory, Mekell, Tigray Ethiopia; 4 Department of Clinical Pharmacy, School of Pharmacy, College of Health Sciences, Mekelle University, Mekelle, Tigray Ethiopia; 5 Department of Medical Microbiology and Immunology, Institute of Biomedical Sciences, College of Health Sciences, Mekelle University, Mekelle, Tigray Ethiopia

**Keywords:** Ocular infection, Bacterial profile, Review

## Abstract

**Background:**

Bacteria are the major contributor of ocular infections worldwide. Ocular infections, if left untreated, can damage the structures of the eye with possible blindness and visual impairments. This work was aimed to review the bacterial profile of ocular infections.

**Methods:**

Literature search was made in different electronic databases; the review was systematically made to get concrete findings.

**Results:**

As far as this review, *Staphylococcus aureus*, Coagulase negative *Staphylococci*, *Streptococcus pneumoniae* and *Pseudomonas aeruginosa* are the leading isolates in ocular infections. Frequent pathogens of the respective clinical diagnose include *Staphylococci*, *Streptococcus pyogenes* and *Pseudomonas aeruginosa* in blepharitis; *Staphylococci*, *Streptococus pneumoniae*, *Pseudomonas aeruginosa*, *Klebsiella pneumoniae* and *Escherichia coli* in Conjunctivitis; *Staphylococci*, *P. aeruginosa* and *E*. *coli* in dacryocystitis; Coagulase negative *Staphylococci*, *Pseudomonas aeruginosa* and *Staphylococcus aureus* in keratitis; *Streptococcus viridians*, *Streptococcus pneumoniae* and Coagulase negative *Staphylococci* in endophthalmitis diagnoses. Endogenous endophthalmitis is associated with *Klebsiella pneumoniae* whereas Coagulase negative *Staphylococci* and *Bacillus spp.* are common causes of post-operative and post-traumatic endophthalmitis. However, the predominant pathogens may not be exactly same in all areas of the world, in the United States for instance, *Staphylococcus aureus*, *Streptococcus pneumoniae* and *Haemophilus influenzae* are the major causes of conjunctivitis.

**Conclusion:**

Gram positive bacteria are the major contributor of bacterial ocular infections. The distribution and proportion of bacterial isolates among clinical diagnoses varied but without exclusive anatomical restriction. To mitigate the burden of bacterial ocular infections, physicians should regard on risk reduction and comply with etiologic approach of diagnosis.

## Background

Bacteria are the major contributor of ocular infections worldwide. Infection can be mono or poly-microbial and is associated with many factors including contact lenses, trauma, surgery, age, dry eye state, chronic nasolacrimal duct obstruction and previous ocular infections [1–3]. Bacteria are generally associated with many types of ocular infections such as conjunctivitis, keratitis, endophthalmitis, blepharitis, orbital cellulitis and dacryocystitis manifestations [4].

Conjunctivitis, inflammation of the mucosa of conjunctiva, is the most frequent ocular case with noticeable economic and social burdens [5]. During chronicity, the disease can affect not only the conjunctiva but also adjacent structures including the eye lid and can be a potential risk for other extra or intraocular infections. Bacteria contribute for about 50–70% of infectious conjunctivitis [6]. Bacterial conjunctivitis is commonly seen in children and the elders but can also be presented among neonates and adults [7, 8].

Blepharitis which is an inflammation of the eyelid can cause loss of eye lash [9]. The infection may not remain localized and is known to spread to other anatomical sites of the eye [10]. Keratitis, the most serious eye infection is the leading cause of corneal blindness. Moreover, the disease can also progress to endophthalmitis if not diagnosed early [11–13].

Exogenous endophthalmitis is an infective complication of primary cataract, intraocular surgery and ocular trauma due to the introduction of infectious pathogens like bacteria whereas the endogenous one is commonly due to systemic dissemination of the pathogens. Both keratitis and endophthalmitis are potentially devastating ocular infections if not diagnosed early [14–16].

Dacryocystitis is an inflammation of the nasolacrimal duct. During chronicity the disease is associated with infection, inflammation of the conjunctiva, accumulation of fluid and chronic tearing. This can be potentially dangerous to ocular tissues such as the cornea; leading to post surgery endophthalmitis [17, 18].

Ocular infections, if left untreated, can damage the structures of the eye leading to visual impairments and blindness. Even though the eye is hard and protected by the continuous flow of tear which contains antibacterial compounds, inflammation and scarring once occurred may not be easily resolved and requires immediate management [10]. Effective management of such infections demands knowledge of the specific etiology. However, ocular infections are mostly managed empirically and little is known about the specific bacterial etiologies [19, 20]. Therefore, the main goal of this work was to review the bacterial profile of the different forms of ocular infections in order to come up with concrete information for physicians and policy makers who deals with ocular infections.

## Methods

The literature search was made in Electronic data base such as PubMed and Google scholars based on the key words of bacterial infection, bacterial profile, microbial spectrum, eye infections and ocular infections. Studies published until 2016 and emphasizing on external and internal ocular infections were subjected to systematic review and those that are close to the review objectives were included from different parts of the world to get concrete findings (76 out of 145); articles written and published in English language, emphasizing on any of the external-ocular infections (dacryocystitis, orbital cellulites, blepharitis, conjunctivitis and keratitis) and internal-ocular (endophthalmitis) infections were included in the study. Articles written other than English language, lacking full length forms and articles published before 1999 were excluded from this review.

## Result

### The distribution and types of bacteria in ocular infections

#### Gram positive bacteria

According to the studies conducted so far Gram positive bacteria are associated with variety of ocular infections. The most commonly reported isolates belong to the genus *Staphylococci* regardless of the study area and population (see Table 1). *Staphylococci* are associated with any type of eye infections including conjunctivitis, blepharitis, endophthalmitis, keratitis, dacryocystitis and orbital cellulitis; most importantly with blepharitis, conjunctivitis and keratitis [10, 21]. Both *S. aureus (Staphylococcus aureus)* and CoNS (Coagulase-negative *Staphylococci*) took the highest proportion of the isolates [2, 22].

**Table 1:** Overall proportion of common bacterial isolates in ocular infection

References, country	% of bacterial isolates										
	Gram positive				Gram negative						
	*S. aureus*	CoNS*	*S. pyogens*	*S. pneumoniae*	*P. aeruginosa*	*E. coli*	*K. pneumoniae*	*Proteus spp*	*H. influenzae*	*Moraxella spp*	*N. gonorrhoeae*
[10], Nigeria	23.7	19.2	6.2	8.6	10.1	4.4	6.2	1.5	7.7	3	3.9
[22], India	13	37			21	3	7				
[21], Ethiopia	28.1	10.1		13.5	20.9	5.4			8.8	4	2.7
[46], India	26.6	6.1	1.6	22.14	8.23	0.9		0.6	3.45	5.4	0.42
[53], Japan	21	31.4		3.2	9.7		2.4	1.6	2.4	0.8	
[50], India	24.6	19.8		4.9	16	12.3	4.9		4.9		
[57], Iran	12.9	32.9		8.6							
[75], India	19.13	20.76	0.55	20.76	4.92	1.1	2.74	0.33		19.13	20.76
[65], Ethiopia	21	27.4	14.5	11.3		8.1	14.5				
[76], India	32.8	39		14.1	3	4.7	6.2				
[77], Ethiopia	21	18.2	4.2	14	4.9	4.9	6.3	3.4	4.2	2.8	
[78], USA	19	25			8	8	10				
[79], Malaysia	17		3.1		16	4	5.5		8.3		
[80], USA	22.1	6.7		2.4	13.7				2.3		
Average	20.1	19.5	4.3	6.7	12	5.9	7.7	1.5	5.3	3.2	2.3

Note: CoNS* = Coagulase Negative *Staphylococci*

Despite their normal existence, CoNS are the most frequent cause of ocular infections with increasing frequencies over time [23]. A 5 year retrospective study in Iran indicated that 40% of infections were due to CoNS [24]. A Similar study in India also found a prevalence of 45.4% [25]. The problem is worse especially in pre-operative and post-operative cases. In a study conducted in patients with cataract surgery, 88.8% of isolates from conjunctival swabs were CoNS [26]. Likewise, 65.9% and 21% of pre-operative cataract patients had CoNS and *S. aureus* isolates respectively. Considering the specific species, *S. epidermidis* and *S. saprophyticus* were the common species of CoNS [27]; both species being dominant in subjects with post-operative endophthalmitis as to the study conducted over 20 years in China [28]. In general *Staphylococcal* infection is common in both post infection and post-operative endophthalmitis cases [29, 30]. Moreover, *S. aureus* and *S. epidermidis* are known to be the common cause of early onset bleb-associated endophthalmitis [31].

*S. aureus* is also the threat of eye infection and has been showing significantly increasing trends over time [32]. Among patients with symptoms of conjunctivitis in Nigeria, it was the leading isolate [2]. Comparable findings were also reported in Ethiopia; *S. aureus* was isolated from 47.6% of blepharitis, 26.6% of conjunctivitis, and 25% of keratitis cases [21]. As in the other clinical cases, it is also common to find both MSSA (Methicillin-sensitive *Staphylococcus aureus*) and MRSA (Methicillin-resistant *Staphylococcus aureus*) in ocular infections. Studies indicated that 47.8% of patients with sight-threatening disorders, 24.5% of lid disorders as well as 10.6% of lacrimal disorders have been infected with MRSA. Hospital acquired MRSA has been involved in Ocular infections in association with health care exposure and ophthalmic surgeries [33, 34]. Tsironi et al. from Greece reported community-acquired MRSA from a young Infant with orbital cellulitis. Upon the microbiological analysis, the isolate was found to be ST80 strain with Panton-valentine leukocidin [35]. Even though the prevalence of *S. aureus* is higher in conjunctivitis diagnoses, MRSA infection was found to be higher in endophthalmitis and keratititis diagnoses [32]. In Taiwan however, MRSA keratitis was 36.1% followed by conjunctivitis 20.1% and endophthalmitis 3.3% [33].

Despite their small proportion, members of the genus *Streptococci* including *S. pneumoniae* (*Streptococcus pneumoniae*), *S. Pyogenes* (*Streptococcus pyogenes*), *Enterococcus* and *S. viridians* (*Streptococcus viridians*) have been involved in ocular infection and had worse outcomes than the *Staphylococcal* infection especially in post-cataract endophthalmitis [4, 36].

Around 80% of *Streptococcal* post injection endophthalmitis cases had final or worse visual outcomes. Moreover, *Streptococcal* infection is 3 times more prevalent in post injection than post operation endophthalmitis suggesting the possibility of contamination by nasopharyngeal floras during the process of injection [16].

*Streptococcal* infection is also common in bleb associated endophthalmitis especially in the early onset bleb-associated endophthalmitis. One study from USA reported that 23% and 65% of isolates from bleb-associated endophthalmitis were *S. pneumoniae* and *S. viridians* respectively while other β-hemolytic *Streptococci* contributed for 12% of the infection [37]. Like *S. aureus* and some gram negatives, *S. pneumoniae* and *S. pyogenes* also reported to causing hospital acquired infections mainly conjunctivitis in neonates with intensive care unit [38]. Even though they are not as common as *S. pneumoniae*, *S. pyogenes*, *S. viridians* and *E. faecalis* (*Enterococcus faecalis*) were also reported in blepharitis, conjunctivitis and keratitis cases [10, 23, 39].

*S. pneumoniae*, the most pathogenic and fastidious *Streptococcus* is among the common isolates in patients with conjunctivitis and dacrocystitis [40–42]. The bacterium is not limited only to these types of infections. Even though it is not as common as CoNS, studies supported the occurrence of the bacteria in eyelid and conjunctival samples of patients with pre-cataract surgery [27]. In a study conducted in India, 12.3% of bacterial keratitis was mainly *Streptococcal* comprised of 11.6% *S. pneumoniae* and 0.6% *S. viridians* [39]. Studies implicated that keratitis due to *S. pneumoniae* is more often associated with permanent loss of vision [43].

Upper respiratory tract infections of *Streptococcal* and *Staphylococcal* origin are sometimes associated with orbital cellulitis and other complications. For instance, orbital cellulitis, endophthalmitis and acute pan-sinusitis was reported as a complication of *Streptococcal* pharyngitis due to *S. pyogenes* [44]. In addition, it is common to find *S. pneuomoniae* and *S. aureus* from specimens of sub periosteal abscess [44]. Because *S. pneumoniae* is a normal inhabitant of the Nasopharynx especially in children, immunization may be needed to reduce the spread to other sites including the eye. Clinical trials in a rabbit model assured that passive immunization can neutralize pneumococcal virulence and remarkably minimize the severity of endophthalmitis [45].

Gram positive bacilli are also known to cause ocular infections. According to the study conducted in Nigeria, Gram positive bacilli accounted for 22.6% of conjunctivitis cases amongst *Corynebacterium* species were predominant followed by *Bacillus* species [2]. *Corynebacterium* infection is higher in blepharitis and conjunctivitis diagnoses; 35.7% and 35.3% of *Corynebacterium* isolates respectively [46]. Not only this, *Corynebacterium* species have also been reported in both acute and chronic dacryocystitis [18]. As compared to the other clinical presentations, Bacillus species are more prevalent in conjunctivitis and post-traumatic endophthalmitis [46, 47]. In addition, both groups have been involved in bacterial keratitis (*Bacillus* species accounted for 5.3% of isolates but 0.3% of the isolates were *Corynebacterium diphtheria*) [39]. Among *Bacillus* species, *B. cereus* (*Bacillus cereus*) is the major one (56.2%) followed by *B. thuringiensis* (*Bacillus thuringiensis*) 26.3%, *B. subtilis* (*Bacillus subtilis*), *B. mycoides* (*Bacillus mycoides*) and *B. pumilis* (*Bacillus pumilis*) 5.2% each; *B .flexus* (*Bacillus flexus*) 2.6% [48]. Less commonly, one study from India reported *Clostridium* species from endophthalmitis cases [49].

### Gram negative bacteria

Studies from different parts of the world indicated that diverse group of Gram negative bacteria are isolated from ocular infections (see Table 1). Among Gram negatives, frequent isolates of conjunctivitis include *P. aeruginosa* (*Pseudomonas aeruginosa*), *E. Coli* (*Escherichia coli*), *Enterobacter* spp. (*Enterobacter cloacae* and *Enterobacter aerogenes*), *C. koseri* (*Citrobacter koseri*), *Proteus* spp., Moraxella spp., and N. gonorrhoeae (Neisseria gonorrhoeae) [2, 38, 50].

In patients presented with dacryocystitis, *Pseudomonas* spp., *Enterobacter*, *K. pneumoniae* (*Klebsiella pneumoniae*) and *H. influenza* (Haemophilus influenza) were reported as the main Gram negative isolates [40]. In addition, another study in Egypt reported isolates of *E. coli* and *A. lwoffii* (*Acinetobacter lwoifi*) from chronic dacryocystitis cases [18].

In keratitis diagnoses, *P. auroginosa*, *E. coli, K. pneumoniae, Acinetobactor,* Serratia (*S. marcescens* and *S. liquefaciens*), *Aeromonas*, *Fusobacterium*, *Enterobactor* spp., *P. mirabilis* (*Proteus mirabilis*), *P. multocida* (*Pasteurella multocida*), *M. catarrhalis* (*Moraxella catarrhalis*) and *H. influenza* have been reported [23, 39, 43]. In addition, *Propiolactone* species have been reported in small prevalence but higher in chronic post-operative endophthalmitis (41–63%). *M. catarrhalis* is also among the common cause of delayed onset bleb-associated endophthalmitis [23, 51]. Unexpectedly, polymicrobial infection was seen in any type of endophthalmitis (post-operative, post-traumatic as well as endogenous); isolates include Serratia spp., *P. aeruginosa, K. pneumoniae, Enterobacter, Acinetobacter,* and *Haemophilus spp.* Moreover, new Gram positive agents (*Pantoea* spp. and *Massilia* spp.) have been reported [49].

*M. catarrhalis* is an opportunistic pathogen which commonly affects immune compromised individuals and alcohol addicts. The organism preferentially affects the eye especially the cornea but rarely other organs. Despite its sluggish microbiological activity it can damage the cornea badly as similar to other virulent pathogens and should be considered as one of the major ocular threats [52]; it results in necrosis and perforation of the cornea due to the deepening keratitis and hyperacute inflammatory reaction. In children, *M. catarrhalis* and *H. influenzae* are common causes of Gram negative bacterial conjunctivitis. However, both species have also been isolated from endophthalmitis and orbital cellulitis cases [53]. According to the retrospective study conducted in India, *M. catarrhalis* (53.17%) and *M. lacunata* (63.83%) infection was significantly higher in blephratis and dacryocytitis diagnoses respectively. Similarly, 73.2% of *H. influenzae* isolates were from conjunctivitis and dacryocystitis diagnoses [46]. The distribution of *H. influenzae* biotypes in ocular diagnoses is noticeably different; biotype II in blepharitis, conjunctivitis, and keratitis; biotype III common in conjunctivitis, keratitis and dacryocystitis; biotype VII only being detected in keratitis diagnoses [54]. *H. influenzae* being the most frequent isolate, small numbers of *H. parainfluenzae* and *H. aegypticus* has also been detected in dacryocystits as to the study from India [46].

N. gonorrhoeae, the Gram negative diplococcic, is the common cause of neonatal conjunctivitis though reported in keratitis cases too. Unlike other cases of conjunctivitis, involvement of this bacterium needs early treatment with topical antibiotics. *N. gonorhoeae* infection happens most commonly vertically. A prospective study in Angola realized that vertical transmission rate of *N. gonorrhoeae* was 50% whereas 10.5% for *M. genitalium* (*Mycoplasma genitalium*) [55]. As far as this review no study reported the occurrence of *N. meningitis* in ocular infections except the one case detected in Nigeria from a child with conjunctivitis and three cases from India [10, 46].

*P. aeruginosa* is the most frequent isolate of Gram negative ocular infections [22]. The percentage of the organism varied among clinical diagnoses of ocular infections but the common cause of bacterial keratitis which is more progressive with large infiltrate and scarring [43, 56]. A study from India supported the significantly higher proportion of the bacteria in dacryocystitis and keratitis manifestations [46]. As to the study conducted in Ethiopia, *P. aeruginosa* isolates were 50% of keratitis diagnoses followed by blepharitis (23.8%), conjunctivitis (11.4%) and blepharoconjunctivitis (16.7%) [21].The reported figure does note that much vary among older and recent works. In Iran for instance, the organism constituted 24.2% of ocular infections [57]. Comparable finding even in studies conducted for extended periods is indicative of the continuous *Pseudomonas* corneal attacks [23]. A 4 years study by Patel et al. convinced the predominance of *P. aeruginosa* in keratitis diagnoses (more than 40%) but 20% in other clinical diagnoses [58]. As to Ly et al. from Australia 21% of isolates from keratitis cases were entirely *P. aeruginosa* [59]. Similarly, in a study conducted in 2014–2015 in Baghdad *P.aeruginosa* was detected in 20% of eye specimens collected from symptomatic patients [60]. Furthermore, a published case report from Spain revealed that *P. aeruginosa* caused ocular necrotizing fasciitis. The diagnosis was made in a 53 old male patient who presented with eyelid edema and purulent secretion in both eyes. The prognosis report indicated that the Infection did get worse and involved pre orbital skin regardless of the antibiotic and surgical managements [61].

Ocular infection by *K. pneumoniae* is most commonly endogenous spread after liver abscess or biliary tract infection. The organism accounts for up to 60% of bacterial endophthalmitis [62]. Endogenous endophthalmitis due to this organism can rapidly cause complete loss of vision [63]. Moreover, studies supported the involvement of the bacterium in any of the other clinical manifestations (blepharitis, conjunctivitis, keratitis and dacryocystitis) (see Tables 2 and 3). Even though *K. pneumoniae* is the most frequent one, *K. oxytoca* (*Klebsiella oxytoxa*) has also been reported in 2% of bacterial keratitis [59].

**Table 2:** Proportion of frequent bacterial isolates in keratitis diagnoses

References, country	% of bacterial isolates									
	Gram positive					Gram negative				
	*S. aureus*	CoNS*	*S. pneumoniae*	*S. viridians*	*Bacillus spp.*	*P. aeruginosa*	*E. coli*	*K. pneuoniae*	*Proteus spp.*	*H. influenzae*
[10], Nigeria	22.4	22.4	3	1.5		22.4	22.4			4.5
[23], UK	14.2	25.8	3	0.4	0.4	24.3		0.4		0.7
[27], Uganda	20.5	66.6	1.3							
[39], India	19.5	20.2	11.6	0.6	5.2	9.7		0.99		
[59],Australia	11	38				21				
[81], Iran	3.85	6.2	24.7	6.6	0.55	24.7	0.55		0.55	
[67], Mexico	7.1	28.1	14.3	14.3		14.3				
[82], Israel	10		14			16				3
[83], France	5.2	32.7	2.3			6.8			0.7	
[84],Thailand	2	11			2	55		2	2	2
[85], Brazil	5.9	4.4	2.4^a^			7.7		0.6	0.6	

Note: *CoNS = Coagulase Negative *Staphylococci;* ^a^ *Streptococus species*

**Table 3:** Comparative distribution of bacterial pathogens in clinical diagnoses other than keratitis

			Type of Clinical diagnose													
			Blephritis		Conjunctivitis							Dacryocystitis		Endophthalmitis		
Reference			[10]	[21] [77]	[2]	[10]	[27]	[38]	[74]	[86]	[69]	[18]	[40]	[37]	[49]	[74]
% 0f bacterial isolates	Gram positive	*S. aureus*	45.5	32	27.1	20.3	21.7	8.2	52.5	5	26	50	19.4			12.5
		CoNS*	22.7	35.6	22.6	18	65		30.1	83.1	45.4	52.2	29		10.5	18.8
		*S. pyogenes*	13.6	3.6		5.7										
		*S. pneumoniae*	9	3.6		10.4	5	1.6					6.5	21	21	31.3
		*S. viridians*	2.3	3.6		4.5				1.7				71	10.5	
		*Enterococcus*				2.25				1.7						
		*Bacillus spp.*								0.9					10.5	
	Gram negative	*P. aeruginosa*		23.8	9.7	8.5		18		2.5	1.6	37.5			21	
		*E. coli*		3.6	6.5	0.45		23		0.9						
		*K. pneumoniae*	6.8			6.8		9.8	2.6	0.4	2.8	17.4	6.5		5.25	12.5
		*Serratia*		4.8				28								
		*Acinetobacter*													5.25	
		*Enterobacter*			1.9			13					6.5			
		*Proteus*		3.6		2.25		1.9	5.14		1.6					6.2
		*H. influenzae*		4.8		10.4							6.5		5.25	
		*M. catarrhalis*		9.5	4.5	4.5				0.4						12.5
		*N. gonorrhoeae*				5.4										

Note: Country of the references: 2 (Nigeria); 10 (Nigeria); 18 (Egypt); 21, 40&77 (Ethiopia); 27 (Uganda); 37 (USA); 38 (Portugal); 49 (India); 74 (Pakistan); 86 (India); 69 (Colombia); *CoNS = Coagulase Negative *Staphylococci*

### Other less commonly encountered and newly reported organisms

Studies implicated that *P. maltocida* has been noticed in a case with purulent conjunctivitis in association with animal contact with a domestic dog being the suspected animal which contaminated an old man after sneezing [64]. Another newly identified organism is *S. maltophilia* (*Stenotrophomonas maltophilia*), an aerobic gram negative bacterium formerly identified as *Pseudomonas maltophilia*. According to the study conducted in China, this organism was detected in 36% of patients with post cataract surgery. Infection was accompanied with complications (retinal detachment and recurrence) and statistically associated with age and posterior capsule rupture [63].

Micrococcus, the Gram positive organism contributed to 4.3% of ocular infections in a study conducted in Iran [57]. Furthermore, a study which targeted endophthalmitis cases has revealed new Gram positive agents including *Lysinebacillus, Gemella and Exiguobacterium* species [49].

## Discussion

The summary of literatures in this review indicated that both gram positive and Gram negative bacteria can cause eye infections. Gram positive bacteria are the leading cause of ocular bacterial infections elsewhere [22, 23, 28, 65]. As compared to Gram positives, Gram negative bacteria are less prevalent but more diverse than Gram positive pathogens [53]. Specifically, *S. aureus*, CoNS, *S. pneumoniae* and *P. aeruginosa* are the leading isolates in ocular infections. Frequent pathogens of the respective clinical diagnose include *Staphylococci*, *S. pyogenes* and *P. aeruginosa* in blepharitis; *Staphylococi*, *S. pneumoniae*, *P. aeruginosa*, *K. pneumoniae* and *E. coli* in Conjunctivitis; *Staphylococci*, *P. aeruginosa* and *E*. *coli* in dacryocystitis; CoNS, *P. aeruginosa* and *S. aureus* in keratitis; *S. viridians*, *S. pneumoniae* and CoNS in endophthalmitis diagnoses. However, there may be some differences in the leading type of bacterial isolates in some parts of the world. In the United States of America for instance, acute conjunctivitis is the most common ocular infection in outpatient healthcare settings and the most common bacterial causes are *S. aureus*, *H. influenzae*, *S. pneumoniae* and *M. catarrhalis* [7, 66]. *S. aureus* is the common cause of conjunctivitis in adults but *S. pneumoniae* and *H. influenzae* are the most frequent causes of bacterial conjunctivitis in Children [66].

The distribution of each bacterial isolate among the different type of ocular infections might be determined by variety of factors. For instance, ocular surface disease and contact lens use have been strongly associated with bacterial keratitis; the inflammatory reaction and anatomical disruption might be a good opportunity for some normal floras such as members of the *Staphylococci* to elicit infection. Moreover, infections might be attributed to traumatic inoculation of the organisms along with foreign bodies and delayed repair secondary to trauma [67]. This has been evidenced in post-traumatic infections of *Staphylococci*, *Streptococci*, *B. cereus* and many other gram negative organisms mentioned in this review [28, 68]. Bacteria such as *P. aeruginosa* are resistant to lens cleaning solutions where they adhere and spread through the formation of lipid rafts in contact lens users [12, 69, 70].

In Individuals with underlying disease such as diabetic mellitus and rheumatoid arthritis, the diminished immunity may result in loss of control of systemic infections with subsequent spread to ocular tissues especially in endophthalmitis [62, 71]. This has also been evidenced in case of *K. pneumoniae* endophthalmitis infections and *M. catarrhalis* keratitis infections [72, 73] In addition, age might be a factor due to waning immunity and susceptibility to bacterial ocular infections [13, 74]. Operative procedures, prophylactic measures and topical use of corticosteroids are also good predisposing factors for bacterial ocular infections associated with the immune suppression and inability to kill some organisms like *P. aeruginosa* and *S. epidermidis* [12, 59]. Because many of the bacteria in this review are nosocomial pathogens, infections might have also been explained by hospitalization and intensive use of medical devices [69]. This has been supported by some other studies in that many organisms including MRSA, *S. marscecence*, *P. aeruginosa* and *E. coli* were associated with hospital acquired neonatal ocular infections [34, 38]. In general, each bacterium can possibly affect the different ocular structures but the frequency of infection largely depends on the type of predisposing factors. This study reviewed the bacterial profile of external-ocular as well as intra-ocular infections; as a limitation *Chylamydia trachomatis* was not included in this review.

## Conclusion

Both Gram positive and Gram negative bacteria are threats of ophthalmic tissues. However, Gram positive bacteria are the major contributor of ocular infections. Bacterial ocular infection involves but is not limited to blepharitis, conjunctivitis, keratitis, endophthalmitis, and orbital cellulitis. The distribution and proportion of bacterial isolates varied among the different clinical diagnoses but without exclusive anatomical restriction. To mitigate the burden of bacterial ocular infections, physicians should regard on risk reduction and comply with etiologic approach of diagnosis.

## Abbreviations

CoNS: Coagulase negative *Staphylococci*
MRSA: Methicillin Resistant *Staphylococcus aureus*
